# The Receptor-Like Kinase SERK3/BAK1 Is Required for Basal Resistance against the Late Blight Pathogen *Phytophthora infestans* in *Nicotiana benthamiana*

**DOI:** 10.1371/journal.pone.0016608

**Published:** 2011-01-27

**Authors:** Angela Chaparro-Garcia, Rachael C. Wilkinson, Selena Gimenez-Ibanez, Kim Findlay, Michael D. Coffey, Cyril Zipfel, John P. Rathjen, Sophien Kamoun, Sebastian Schornack

**Affiliations:** 1 The Sainsbury Laboratory, John Innes Centre, Norwich, United Kingdom; 2 John Innes Centre, Norwich, United Kingdom; 3 Department of Plant Pathology and Microbiology, University of California Riverside, Riverside, California, United States of America; University of Wisconsin-Milwaukee, United States of America

## Abstract

**Background:**

The filamentous oomycete plant pathogen *Phytophthora infestans* causes late blight, an economically important disease, on members of the nightshade family (Solanaceae), such as the crop plants potato and tomato. The related plant *Nicotiana benthamiana* is a model system to study plant-pathogen interactions, and the susceptibility of *N. benthamiana* to *Phytophthora* species varies from susceptible to resistant. Little is known about the extent to which plant basal immunity, mediated by membrane receptors that recognise conserved pathogen-associated molecular patterns (PAMPs), contributes to *P. infestans* resistance.

**Principal Findings:**

We found that different species of *Phytophthora* have varying degrees of virulence on *N. benthamiana* ranging from avirulence (incompatible interaction) to moderate virulence through to full aggressiveness. The leucine-rich repeat receptor-like kinase (LRR-RLK) BAK1/SERK3 is a major modulator of PAMP-triggered immunity (PTI) in *Arabidopsis thaliana* and *N. benthamiana*. We cloned two *NbSerk3* homologs, *NbSerk3A* and *NbSerk3B*, from *N. benthamiana* based on sequence similarity to the *A. thaliana* gene. *N. benthamiana* plants silenced for *NbSerk3* showed markedly enhanced susceptibility to *P. infestans* infection but were not altered in resistance to *Phytophthora mirabilis*, a sister species of *P. infestans* that specializes on a different host plant. Furthermore, silencing of *NbSerk3* reduced the cell death response triggered by the INF1, a secreted *P. infestans* protein with features of PAMPs.

**Conclusions/Significance:**

We demonstrated that *N. benthamiana* NbSERK3 significantly contributes to resistance to *P. infestans* and regulates the immune responses triggered by the *P. infestans* PAMP protein INF1. In the future, the identification of novel surface receptors that associate with NbSERK3A and/or NbSERK3B should lead to the identification of new receptors that mediate recognition of oomycete PAMPs, such as INF1.

## Introduction

The first line of host defence against pathogenic organisms consists of surface-exposed pattern-recognition receptors that mediate the recognition of highly conserved microbial molecules [1]–[2], termed pathogen-associated molecular patterns (PAMPs). Examples of PAMPs recognised in plants are peptides derived from the bacterial flagellin and elongation factor Tu (EF-Tu), as well as several conserved secreted proteins from bacteria, fungi and oomycetes, and the polysaccharides chitin and beta-glucans [3].

PAMP triggered immunity (PTI) in plants is thought to be the main mediator of basal immunity [4]. PTI is mediated by peripherally located receptor-like proteins (RLPs) or receptor-like kinases (RLKs) which consist of extracellular repeats that are linked by a transmembrane domain to either an intracellular adapter domain (RLPs) or a kinase domain (RLKs) [5]. In Arabidopsis plants, the leucine-rich repeat (LRR)-RLK FLS2 (Flagellin Sensing 2) was shown to heterodimerise with the regulatory LRR-RLK BAK1 upon binding of the cognate PAMP leading to activation of signal transduction [6], [7]. BAK1 is also required for responses to other PAMPs [5], [7], [8]. Arabidopsis BAK1 (also called SERK3) is a member of a family of five somatic embryogenesis receptor kinases (SERKs) [9], which are important regulators for RLKs involved both in immune responses and in various developmental processes. SERKs consist of five extracytoplasmic LRRs, a family specific serine-proline-rich hinge region, a transmembrane domain, a cytoplasmic Ser/Thr kinase and a C-terminal tail [10]. BAK1/SERK3 function appears to be conserved in solanaceous plants, such as tobacco and tomato [7], [11], [12], however, no corresponding full-length coding sequences have been described to date.

Plants of the nightshade family (Solanaceae), particularly the crop plants potato and tomato, are infected by the economically important filamentous pathogen *Phytophthora infestans*, the causal agent of late blight disease. *P. infestans* causes disease on a range of solanaceous species including, but not limited to, tomato, potato and the wild tobacco-relative *Nicotiana benthamiana* [13], [14]. Similar to other plant pathogens, *P. infestans* is thought to colonize these host plants by suppressing basal immunity through the production of a wide array of effector proteins [15], [16]. However, some plants such as tobacco (*Nicotiana tabacum*) are resistant to *P. infestans* [17], possibly because of the recognition of PAMPs such as the secreted protein INF1, and/or the inability of *P. infestans* to suppress immunity on this plant. INF1 recognition results in localised plant cell death (hypersensitive response) and prevents pathogen growth [17]. Heese and co-authors (2007) showed that INF1 also elicits a cell death response and triggers accumulation of reactive oxygen species (ROS) in *N. benthamiana*. This cell death was abrogated upon knock-down of *Serk3*-like sequences [7]. INF1 has, therefore, emerged as a typical PAMP given that it is widely conserved in *Phytophthora* and *Pythium*, and triggers defense reponses dependent on a conserved immune regulator that are suppressed by the effector AVR3a and two other RXLR-type effectors of *P. infestans* [7], [18], [19], [20], [21]. Nevertheless, the nature of the INF1 receptor and the composition of the receptor complex remain unknown, although INF1-interacting plant membrane proteins have been identified [22].

*Nicotiana benthamiana* is a model plant for studies of host-pathogen interactions [23]. Initially, it was used extensively in virus research because it is unusually susceptible to many virus species. More recently, *N. benthamiana* has also emerged as a popular model for the study of bacterial plant pathogens and filamentous pathogens such as fungi and oomycetes. In the oomycete community, *N. benthamiana* is utilised as a model system to study *P. infestans* pathogenicity and host-interactions at both functional and cellular levels [13], [14], [18], [19]. *Nicotiana benthamiana* has several advantages over potato and tomato as an experimental system to study *P. infestans* pathogenicity; particularly the facile application of transient gene expression and gene silencing assays [23]. Moreover, *N. benthamiana* is also emerging as an excellent system for microscopic studies of infections caused by filamentous plant pathogens, such as *Phytophthora* spp., given that tissues can be mounted and analysed without prior treatment [24], [25].

The aim of the present study was to address the extent to which defence responses mediated by NbSERK3 contribute to resistance to *P. infestans* in *N. benthamiana*. We found that four species of *Phytophthora* have different degrees of virulence on *N. benthamiana* ranging from avirulent *P. mirabilis* to moderately virulent *P. infestans* through to full aggressive *P. capsici*. We identified and silenced the expression of two *N. benthamiana* orthologs of the *Arabidopsis thaliana* gene *BAK1/SERK3*. Remarkably, *NbSerk3* silencing resulted in significantly enhanced susceptibility to *P. infestans* but did not affect resistance to *Phytophthora mirabilis*, a sister species of *P. infestans*. NbSERK3A and NbSERK3B were also shown to regulate plant responses triggered by the *P. infestans* PAMP protein INF1.

## Results

### *N. benthamiana* shows varying degrees of susceptibility to *Phytophthora* species and *P. infestans* isolates

**Figure 1:**
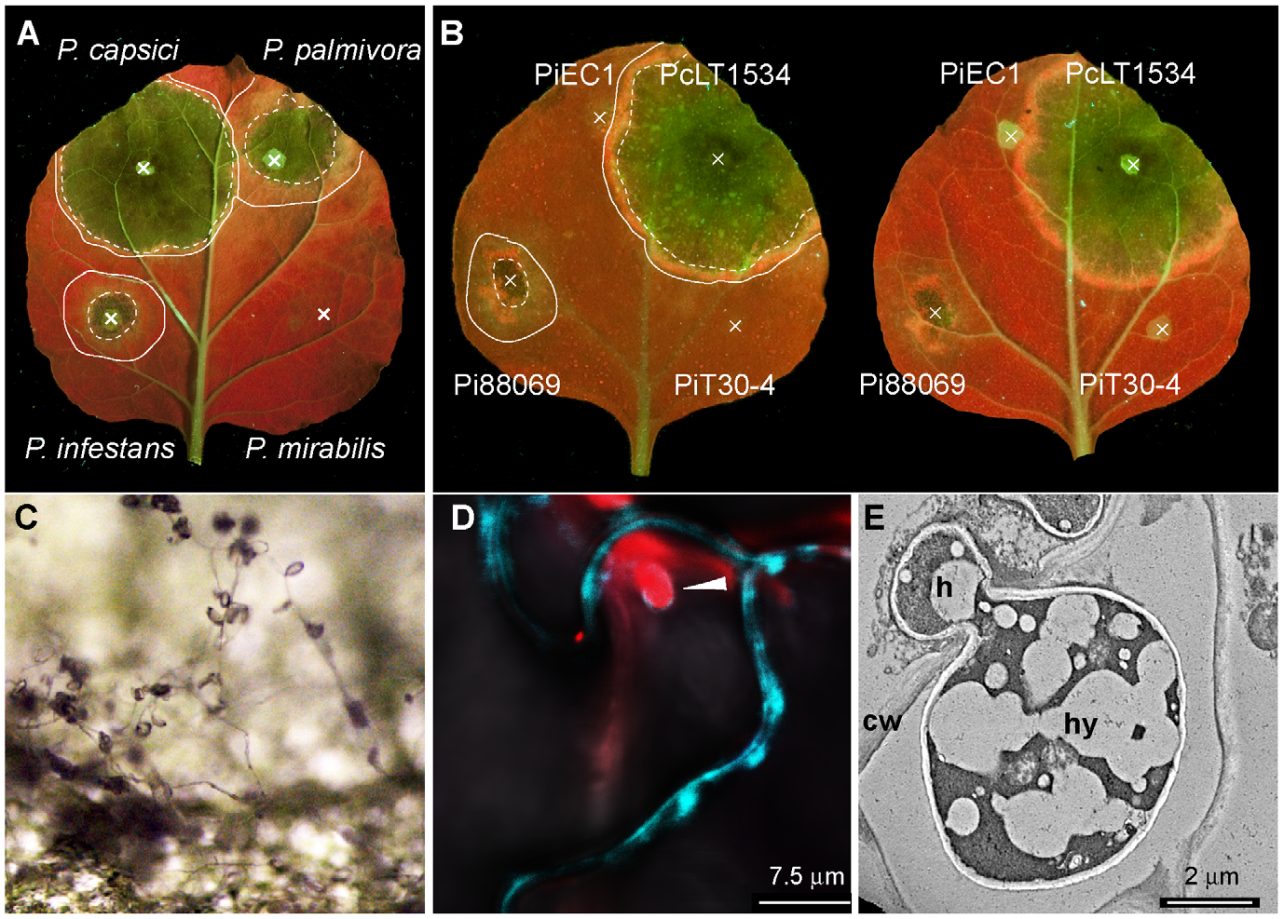
*N. benthamiana* shows varying degrees of susceptibility to *Phytophthora* species and *P. infestans* isolates. Detached leaves of *N. benthamiana* were infected with spore solution droplets (marked as X) of *Phytophthora capsici* LT1534, *P. palmivora* 16830, *P. infestans* 88069 and *P. mirabilis* PIC99114. (A) or *P. infestans* isolates (B) and using *P. capsici* as a reference. Photographs were taken 3 days post inoculation under UV illumination. Lines mark infected areas, dotted lines mark the border between necrotic tissue and biotrophic tissue. *P. infestans* is able to produce sporangia on *N. benthamiana* 8 days post infection (C*). P. infestans* isolate 88069td (expressing tdTomato red fluorescent protein) formed digit like haustoria (arrowhead) that invaginated the *N. benthamiana* cell membrane labelled by transient *Agrobacterium tumefaciens* mediated expression of a membrane localised cyan fluorescent protein at 3 dpi (D). Haustoria were also observed by electron microscopy (E). h, haustorium; cw, cell wall; hy, hypha.

We wanted to compare different *Phytophthora* species and isolates of *P. infestans* in their ability to colonize *N. benthamiana* leaves. We infected 25-day-old detached *N. benthamiana* leaves with zoospore suspensions of isolates representing four different species and monitored infection using ultraviolet (UV) illumination. We could discriminate two circular zones of infection; a central necrotic area around the inoculation site which fluoresced green under UV, and a ring of yellow fluorescent tissue that did not show macroscopic cell death and corresponds to the biotrophic phase of the disease (Fig. 1A,B). Zone diameters indicate that the tested *P. capsici* and *P. palmivora* isolates are highly virulent with large infection site diameters, while *P. infestans* isolate 88069 showed smaller infection zones (Fig. 1A). On the other hand, *P. mirabilis* did not cause spreading lesions on *N. benthamiana*, instead triggering a localized cell death response typical of the hypersensitive response (HR) (Fig. 1A). We consistently observed a larger autofluorescent ring with *P. palmivora* and *P. infestans* infections compared to *P. capsici* infections, suggesting differences in the extent of the biotrophic phase between these pathogens. In summary we found varying degrees of infection phenotypes ranging from complete HR-based resistance (*P. mirabilis*) to very susceptible (*P. capsici*).

To determine to what extent moderate colonisation of *N. benthamiana* by *P. infestans* is isolate-specific, we tested two additional isolates (T30-4 and EC1). Three days post-inoculation (dpi), spreading infections of *P. capsici* and to a lesser extent *P. infestans* 88069 were observed (Fig. 1A–C). However, no watersoaking or growing necrosis was observed with the isolates EC1 and T30-4. Instead, strong UV autofluorescent accumulation of defence compounds was observed in proximity of the spore droplet (Fig. 1B), suggesting a defence response that limits infection as reported earlier [17]. At later stages sporangia formed by strain 88069 were observed (Fig. 1C) and viable, infectious zoospores could be obtained from them (data not shown). Furthermore, confocal fluorescence microscopy and electron microscopy revealed haustorial structures between 2–5 dpi upon infection with *P. infestans* 88069 or the derived strain 88069 (tdtomato) that expresses a red fluorescent protein (Fig. 1D,E). Notably, EC1 and T30-4 isolates showed few intercellular hyphae upon microscopical inspection, but only at 4–5 dpi (data not shown).

These data suggest that of all tested *P. infestans* isolates, 88069 most successfully infects and completes the asexual life cycle on *N. benthamiana* plants, while the other isolates are less aggressive and trigger a stronger defence response. We hypothesized that some of the observed isolate variation in infection efficiency is driven by varying degrees of suppression of PTI.

### Identification of *Nicotiana benthamiana* homologs of Arabidopsis BAK1

SERK3 (also termed BAK1) is a member of the family of five SERK proteins in Arabidopsis. Previous work points to a role of *N. benthamiana* SERK3 homologs in immune responses towards the oomycete pathogen *Hyaloperonospora arabidopsidis* [7]. Heese et al (2007) also showed that expression of *SERK3* homologs is required for multiple PAMP-mediated responses in *N. benthamiana*, thereby suggesting functional and sequence conservation of SERK3.

We thus wanted to address whether knockdown of NbSERK3 expression enhances *N. benthamiana* susceptibility to *P. infestans*. To date, no full length homolog of SERK3/BAK1 from *N. benthamiana* has been reported. Searches in the expressed sequence tag (EST) databases of *Nicotiana* species revealed only a partial *Serk3* -like sequence from tobacco, while all other ESTs with extended similarity to *BAK1/SERK3* did not identify Arabidopsis SERK3 in reciprocal BLAST analyses and are most likely not functional homologs of SERK3 (see methods).

**Figure 2:**
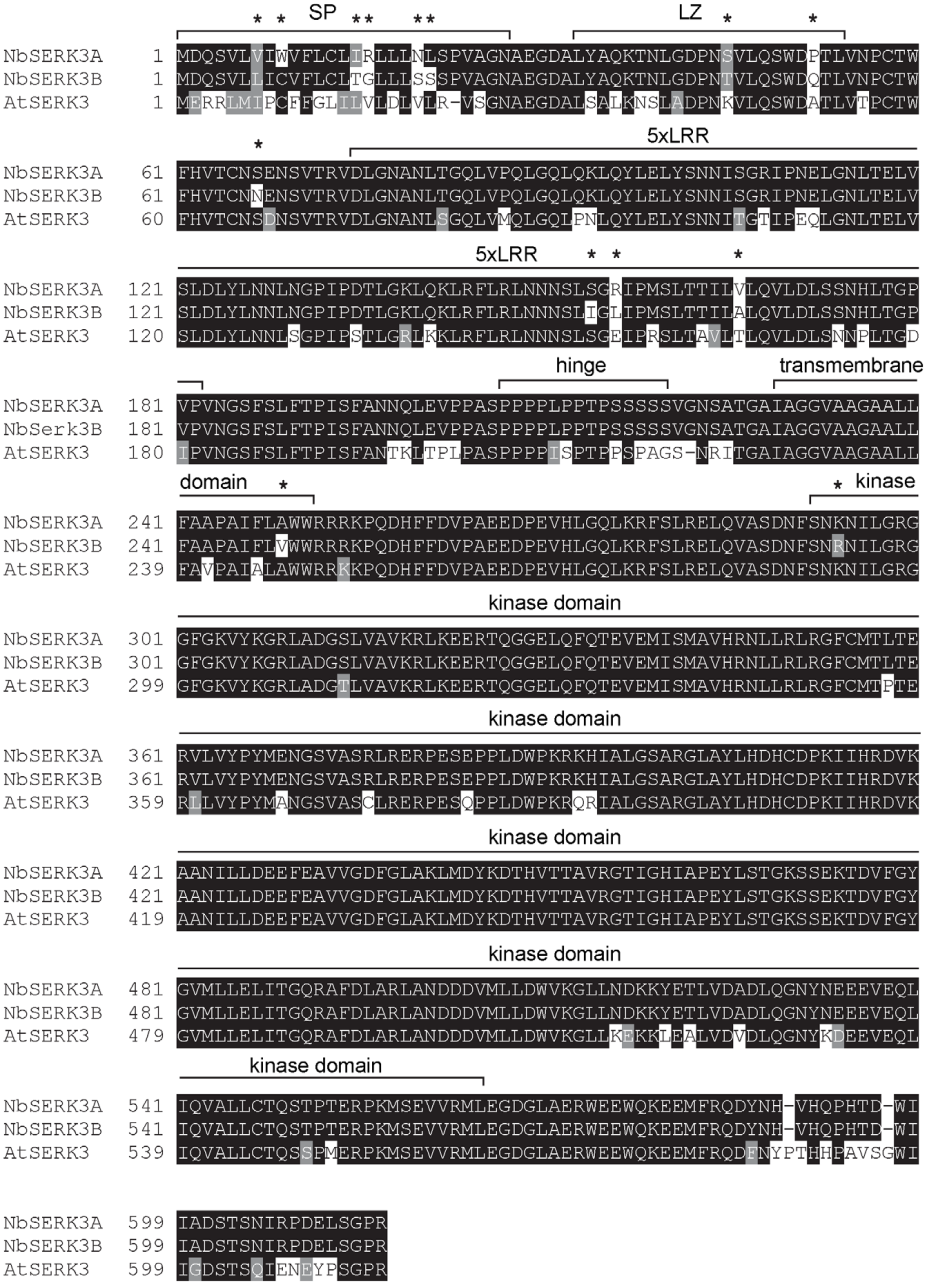
*N. benthamiana* homologs of AtBAK1. ClustalW alignment of SERK3 homologs from Arabidopsis and *Nicotiana benthamiana*. Amino acid residues are shaded black if identical or gray if similar. The signal peptide was predicted using SignalP3 web prediction tool (see methods). Domain identities are labelled above sequences. Asterisk indicates difference between NbSERK3A and NbSERK3B.

To facilitate the cloning of *Serk3* homologs from *N. benthamiana* using conserved sequences, we screened the genomes of the related solanaceous species, tomato and wild potato (*Solanum phureja*), which identified two *Serk3* homologs from tomato (*SlSerk3A*, *SlSerk3B*) and one from *S. phureja* (*SpSerk3A*). Based on these sequences we then cloned two *NbSerk3* homologs (*NbSerk3A*, *NbSerk3B*) from *N.benthamiana* cDNA using conserved primers (see methods). Reciprocal BLAST with *NbSerk3A* and *NbSerk3B* against Arabidopsis identified BAK1/SERK3 as the top hit, suggesting that the identified sequences were the most similar homologs. The coding sequences of *NbSerk3A* and *NbSerk3B* differ in 22 single nucleotide polymorphisms (98.8% identical). Both derived NbSERK3 proteins follow the conserved structure consisting of an N-terminal signal peptide, followed by four equally spaced leucines that could form a leucine zipper (LZ), five extracellular leucine rich repeats (LRR), a proline/serine rich hinge domain, a transmembrane domain and an intracellular serine/threonine kinase domain (Fig. 2). Notably, 12 of 14 amino acid polymorphisms between NbSERK3A and NbSERK3B are located in extracytoplasmic domains. In summary, we identified two close *N. benthamiana* SERK3 paralogs, highly homologous to Arabidopsis BAK1/SERK3, which are conserved in sequence and domain structure.

### *NbSerk3* silencing in *N. benthamiana* results in enhanced susceptibility to *P. infestans*

**Figure 3:**
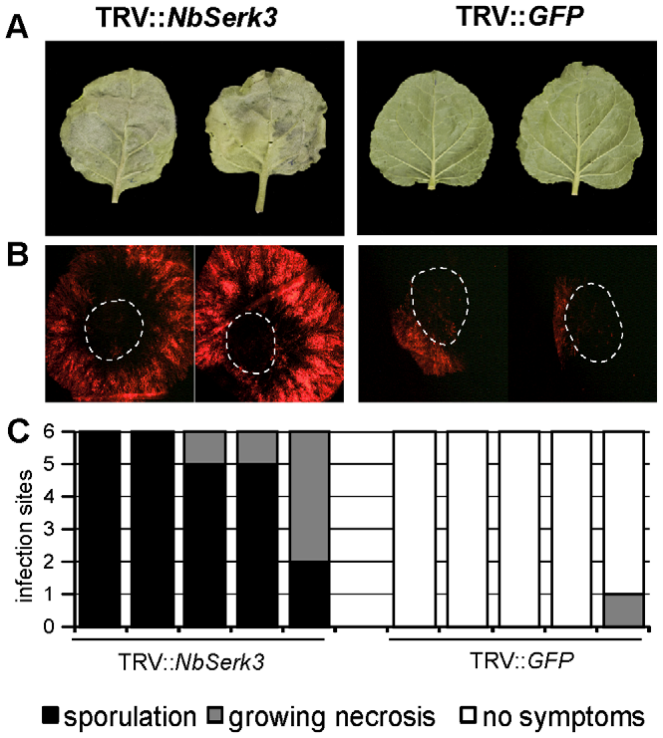
*NbSerk3* silenced *N. benthamiana* shows enhanced susceptibility to *P. infestans* infection. *N. benthamiana* plants were silenced using tobacco rattle virus vectors harbouring an empty cloning site (TRV::*GFP*) or a partial *NbSerk3* sequence (TRV::*NbSerk3*, [7], see also Fig. S1). Nineteen days later, leaves were detached and spore droplet inoculated within the dotted lines with *P. infestans* 88069 (A) or 88069td (B). Pictures were taken 6 dpi (A) and 3 dpi (B). Infection stages of at least 5 independent plants of each silencing construct were scored 4 dpi (C).

We next asked whether silencing of *NbSerk3* variants affects colonisation of *N. benthamiana* by *P. infestans*. Plants were silenced using virus-induced gene silencing (VIGS) and the constructs TRV::*GFP* or TRV::*NbSerk3* [7]. Silencing was confirmed by RT-PCR using a primer combination that annealed to both *NbSerk3* transcripts, upstream of the region targeted by the TRV::*NbSerk3* silencing construct (Fig. S1). Detached leaves of silenced plants were challenged 19 days after silencing by drop inoculation with *P. infestans* zoospore solutions of strains 88069 or the red fluorescent 88069td. During infection we repeatedly observed better infection and faster progression at the infection sites to the stage of sporulating hyphae in several independently *NbSerk3*-silenced leaves, alongside an extended hyphal growth (Fig. 3). By 4–5 days whole leaves were infected and sporulating, while no sporulation was observed on the control silenced leaves (Fig. 3E). Similar results were obtained with two other *P. infestans* isolates (Fig. S2), indicating that *NbSerk3* silencing enhanced the susceptibility of *N. benthamiana* to *P.infestans* infection.

### Silencing of *NbSerk3* in *N. benthamiana* does not alter resistance to *P. mirabilis*

**Figure 4:**
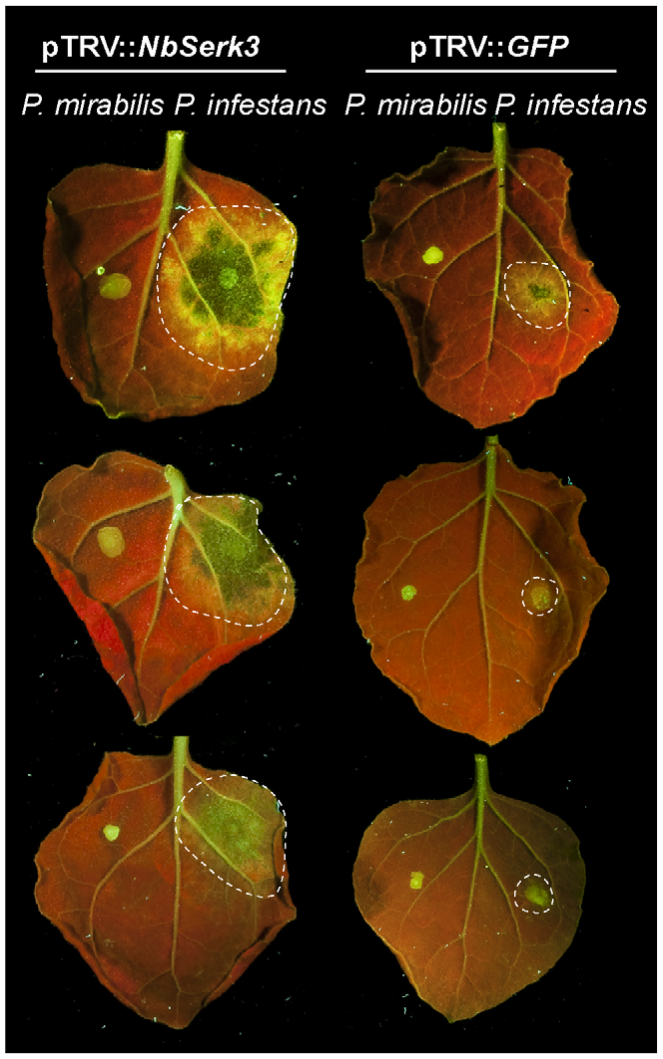
*NbSerk3* -silenced *N. benthamiana* leaves are not infected by the non-host pathogen *P. mirabilis.* * NbSerk3* -silenced (left) or control silenced plants (right) were inoculated with *P. mirabilis* (left leaf halves) or *P. infestans* 88069 (right leaf halves). Images were taken three dpi with UV illumination. Dotted lines represent infected areas. Note the occurrence of autofluorescent spots at *P. mirabilis* infection sites.

To study whether NbSERK3A and NbSERK3B are also required for resistance towards *P. mirabilis* we carried out zoospore drop inoculations of *NbSerk3-* or control silenced leaves as described above. We did not observe any difference in infections with *P. mirabilis* between *NbSerk3*-silenced and control leaves, while *P. infestans* infections were enhanced (Fig. 4). UV autofluorescence within the leaf area under the droplet suggested a localised defence response (Fig. 4). Drop inoculations on the host plant *Mirabilis jalapa* showed sporulating infections 3–4 days post spore inoculation and confirmed viability of the *P. mirabilis* zoospores (data not shown). This data suggest that other mechanisms besides NbSERK3-mediated defence response contribute to resistance towards *P. mirabilis* infection.

### INF1 purified from *P. infestans* triggers cell death and a late ROS burst in *N. benthamiana*

A well-studied elicitor of *P. infestans* that is recognised by *N. benthamiana* is the INF1 protein, which is secreted by the pathogen into the extracellular space. Heese et al. (2007) established a link between INF1 responses (cell death, ROS burst) and *NbSerk3* mainly using recombinant INF1 protein produced by *E.coli* (INF1[Ec]).

**Figure 5:**
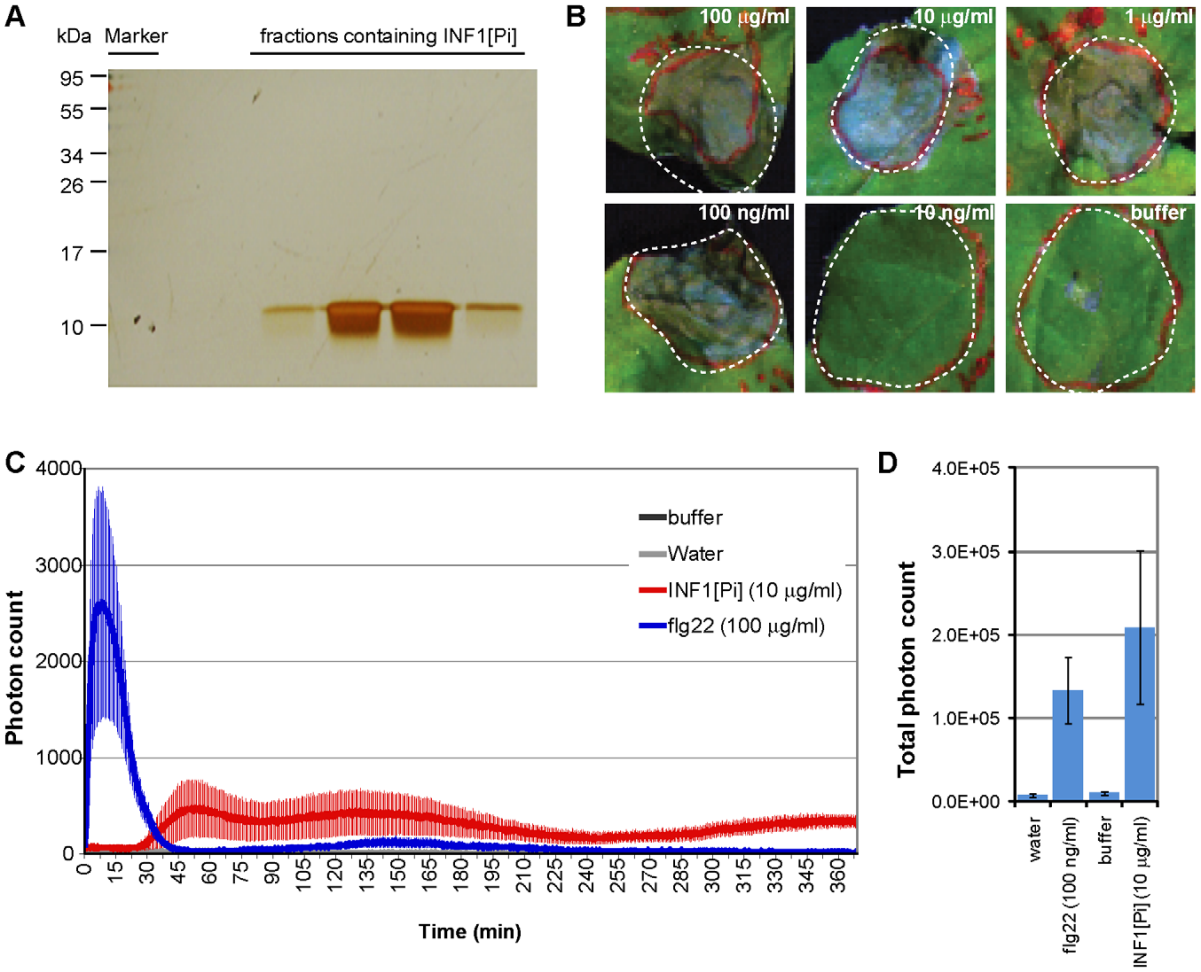
INF1 purified from *P. infestans* triggers cell death and a delayed ROS burst in *N. benthamiana.* INF1[Pi] was purified from *P. infestans* 88069 culture supernatant and fractions loaded on a silver stained gel to confirm absence of contaminating proteins (A). Cell death inducing activity in *N. benthamiana* was tested by infiltration of stepwise diluted INF1[Pi] protein solution or buffer into the plant apoplast and pictures were taken 6 dpi (B). Incubation of leaf discs in INF1 or flg22-containing buffer triggered accumulation of ROS, measured in a peroxidase assay as emitted photons. Accumulation of ROS is shown over time (C) or as total count (D).

To expand these results and to exclude modulation of PAMP responses by residual *E. coli* PAMPs that induce SERK3-dependent responses, we used INF1 purified from *P. infestans* (INF1[Pi]). To that end, we purified INF1[Pi] from *P. infestans* strain 88069 culture supernatant using anion exchange chromatography. INF1 elution fractions did not show additional protein bands in silver-stained protein gels (Fig. 5A).

Similar to the data obtained with INF1[Ec] we found that INF1[Pi] protein infiltration into *N. benthamiana* induced cell death, suggesting that the purification process did not affect its known activity (Fig. 5B).

We then tested 10 µg/ml of purified INF1[Pi] for its ability to trigger a temporal accumulation of ROS. We observed a significant ROS burst peaking at 50 min post-application of INF1. Notably, the burst was lower and delayed compared to flg22-triggered ROS burst peaks in *N. benthamiana* (Fig. 5C). However total photon counts were comparable between flg22 and INF1 response (Fig. 5D). This indicates that INF1-triggered ROS response differs in kinetics and amplitude from the flg22-triggered response without affecting the total amount of produced ROS.

### Cell death triggered by INF1 protein purified from *P. infestans* requires NbSERK3

**Figure 6:**
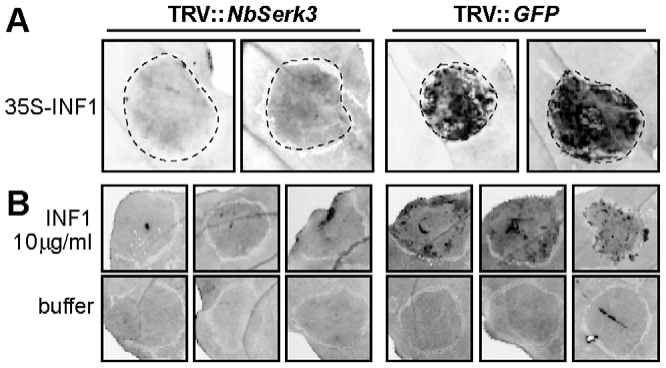
Cell death triggered by INF1 in *N. benthamiana* requires NbSERK3 variants. Leaves of *NbSerk3* or control silenced plants were infiltrated with *A. tumefaciens* harbouring 35S-INF1 for transient *in planta* expression (A) or with *P. infestans* purified INF1 (B). Phenotypes were scored 6 dpi by visualizing cell death (dark areas) using UV trans-illumination.

To address whether NbSerk3 variants are required for INF1[Pi]-triggered responses, we silenced *NbSerk3A*/*B* using VIGS. Plants were either infected with TRV::*GFP* (negative control) or TRV::*NbSerk3*. Nineteen days later, leaves were infiltrated with *A. tumefaciens* harbouring *INF1-*expressing T-DNA constructs, or with purified INF1[Pi]. We observed a cell death response with *in planta* expressed INF1 and 10 µg/ml INF1[Pi] in control plants. Interestingly, plants silenced for *NbSerk3*A and *NbSerk3B* showed a significantly reduced cell death response to *35S-INF1* and injected INF1[Pi] (Fig. 6). We conclude that *NbSerk3A* and *NbSerk3B* are required for cell death triggered by the secreted protein INF1 from *P. infestans*.

## Discussion

In this study we show that different species of *Phytophthora* have varying degrees of virulence on *N. benthamiana* ranging from incompatible to moderate virulence through to full aggressiveness. Three of the tested species, *P. infestans*, *P. palmivora*, and *P. capsici*, can colonise *N. benthamiana*, while a fourth species *P. mirabilis* was avirulent. This suggests that this model plant species can be used to study a whole range of *Phytophthora*-host interactions. We further characterised the moderate virulence of *P. infestans* by testing the hypothesis that surface immunity is implicated. To this purpose, we identified two *NbSerk3* genes from *N. benthamiana* that are similar to the key regulator of surface immune receptors BAK1 of Arabidopsis. *NbSerk3* silencing in *N. benthamiana* resulted in markedly enhanced susceptibility to *P. infestans* but did not alter resistance to *P. mirabilis*. Silencing of *NbSerk3* also reduced the cell death response triggered by INF1 protein purified from *P. infestans*. We conclude that *N. benthamiana* exhibits an effective basal resistance against *P. infestans*, probably via recognition of the INF1 protein, resulting in a relatively moderate degree of compatibility. Overall, these results further confirm the general importance of PTI in plant-pathogen interactions and suggest that the concepts developed for bacterial pathogens can be extended to oomycetes [26].

There are seemingly conflicting reports in the literature on the extent to which *N. benthamiana* is susceptible to *P. infestans*. An early report by Kamoun *et al*. [17] concluded that *P. infestans* is avirulent on *N. benthamiana*, and that resistance is partially due to recognition of the elicitin protein INF1. However, the authors noted the occurrence of secondary hyphae and haustoria on *N. benthamiana* although they were limited to the initial infection zone [17]. Later reports, including field and greenhouse trials with *N. benthamiana*, concluded that this plant is susceptible to several *P. infestans* isolates and that compatible interactions can be established [13], [27], [28]. More recently, Shibata et al. [14] confirmed that *N. benthamiana* is susceptible but that the age of the host plant affects the ability of *P. infestans* to infect. In this study, we confirmed that *P. infestans* can establish spreading infections on *N. benthamiana* that are not limited to the initial inoculation site (Fig. 1 and 3). We conclude that many *P. infestans* isolates, including 88069, can colonize *N. benthamiana*. However, *P. infestans* shows clearly reduced levels of virulence relative to *P. palmivora* and *P. capsici*.

We found that the aggressiveness of *P. infestans* was dramatically enhanced upon silencing of *NbSerk3* in *N. benthamiana* (Fig. 3), suggesting that variation in virulence between isolates of *P. infestans* may depend on suppression of NbSERK3-mediated immunity, possibly by host translocated RXLR effectors such as AVR3a, PEXRD8, and PEXRD39 which were shown to suppress INF1-triggered cell death [19], [20]. Conversely, NbSERK3-mediated immunity is not required for the effective resistance of *N. benthamiana* to *P. mirabilis* (Fig. 4). This NbSERK3-independent resistance could be due to the recognition by *N. benthamiana* of specific effector proteins by host disease resistance proteins. Indeed, Wei et al. showed that recognition of a single effector protein, HopQ1-1, was sufficient to define host-specificity in the interaction between *P. syringae* DC3000 and *N. benthamiana* [29].

How does *P. infestans* suppress immunity to successfully colonize *N. benthamiana*? Most likely this involves host-translocated effectors, such as members of the RXLR family [30], [31]. For instance, the RXLR effector AVR3a targets the E3 ubiquitin ligase CMPG1 to suppress defence during the biotrophic stage of the late blight disease [18]. CMPG1 is a component of membrane receptor-mediated immunity to several pathogens [18], [32]. Interestingly, both NbSERK3 (Fig. 6) and CMPG1 are required for responses to INF1, when this protein is delivered by infiltration into the apoplast of *N. benthamiana* [7], [32]. In the future, identification of a membrane receptor for INF1 and unravelling its interplay with the genetic elements *CMPG1*, *NbSerk3A* and *NbSerk3B* will help in understanding how *P. infestans* suppresses surface immunity and whether these elements are targeted by dedicated *P. infestans* effectors.

We discovered that *N. benthamiana* carries two *NbSerk3* variants unlike Arabidopsis, which only harbours a single *BAK1/SERK3* gene. Most likely, this can be attributed to the allopolyploidic nature of *N. benthamiana* [23]. It remains to be determined whether both *NbSerk3* homologs contribute to the same extent to basal resistance to *P. infestans*. The two *NbSerk3* genes are too similar to enable specific silencing of individual copies and in our experiments transcript level reduction was observed for both *NbSerk3* variants (Fig. S1). Interestingly, we also identified two sequences similar to *BAK1/SERK3* in the recently sequenced genome of tomato while only one copy was detected in the genome of wild potato *Solanum phureja* (see Methods). It remains to be determined whether the copy number variation of the *Serk3* genes in solanacaeous plants is biologically relevant. However, knock-down experiments suggest that the SERK3 proteins are important elements of surface immunity in the Solanaceae [7], [11], [12].

We noted that differences between the NbSERK3 variants almost exclusively reside in the extracytoplasmic domains (Fig. 2). By analogy with BAK1, NbSERK3 hetero-oligomerisation with other RLKs is likely mediated by the kinase domain-containing C-terminus while downstream signalling is mediated by the trans-interacting partner RLK [10], [33]. Less is known about the role of the extracytoplasmic domains of BAK1/SERK3 in interaction with the ligand-binding RLK or signalling. Mutations of Arabidopsis BAK1 LZ domain residues L32E and L46E, which are conserved in the NbSERK3 variants, affects heteromerisation with BRI1 in yeast two-hybrid assays but alterations in the subcellular localisation or stability of the mutant BAK1 were not addressed [33], [34].

Further comparison of the NbSERK3 proteins to Arabidopsis BAK1 revealed significant divergence within the signal peptide, the proline/serine-rich hinge region and the non-kinase C-terminus (Fig. 2). Remarkably, the region between the transmembrane and kinase domains, which is often highly variable in LRR-RLKs, is conserved between all three proteins with only one conservative lysine/arginine substitution. By analogy with the mammalian receptor EGFR this juxta-membrane region may contribute to heteromerisation with other RLKs [35]. Inspection of known BAK1/SERK3 phosphorylated residues [33] showed that both activating and suppressory phosphorylation sites are conserved between BAK1 and NbSERK3. A threonine to serine exchange at position 312 (based on BAK1 sequence) should not affect the phosphorylation of this residue. The extent to which other identified polymorphisms affect the activities of SERK3 remains to be addressed. Such analyses could include the effect on downstream signalling or interactions with *Nicotiana*-specific RLKs or RLPs. BAK1/NbSERK3 chimeric constructs might help to dissect the contribution of specific domains as performed previously with FLS2, EFR and the wall-associated kinase WAK1 [36], [37], [38]. In summary, the availability of the NbSERK3 proteins enables additional structure/function studies of this important immune regulator and may also lead to the identification of additional PAMP receptors recognizing oomycete pathogens.

### Conclusion

NbSERK3A and NbSERK3B contribute to basal immunity and INF1-mediated recognition. They provide a tool to study PTI signalling in *N. benthamiana* and reveal that the concepts developed for bacterial pathogens can be extended to oomycetes.

## Materials and Methods

### EST database searches

The TIGR EST database (http://compbio.dfci.harvard.edu/cgi-bin/tgi/Blast/index.cgi) was searched using tBlastN and AtBAK1 as a query with species selection for *N. benthamiana*; pepper; petunia; potato; tobacco; tomato. Four ESTs with similarity to AtBAK1 extending over the kinase domain were identified (potato|TC194641, tobacco|TC102165, tobacco|TC84094, potato|TC201408). However, reciprocal BlastX against Arabidopsis only confirmed the tobacco ortholog |TC102165 as being truly orthologous to Arabidopsis *BAK1/SERK3*. In addition, the EST identified did not represent a full length *Serk3* coding sequence. The potato|TC194641 appeared orthologous to Arabidopsis *SERK2*, the tobacco|TC84094 to *FEI2* and the potato|TC201408 to *NIK3*.

### Cloning of *NbSerk3A* and *NbSerk3B*

tBlastN searches using AtBAK1 against tomato and potato genomes identified two tomato genomic scaffolds encoding SERK3 homologs in the sequenced tomato genome (http://www.solgenomics.org) and one genomic scaffold encoding a *Solanum phureja* (http://www.potatogenome.net) *Serk3* homolog.

GeneWise alignment of the Arabidopsis BAK1/SERK3 amino acid sequence to the genomic sequences identified above was used to detect exon-intron structures and to derive the putative coding sequence. Secretion signals for all SERK3 homologs were predicted using SignalP3. We derived conserved primers (SolSERK3-UTR5F/Seb: 5′-GGTGGTTTCAATGAA GAATCTTGGTTTTTAGTTG-3′, SolSERK3-UTR3R/Seb: 5′-GTAATGACCCAATCACCTATACACATTTGGAC-3′) from untranslated regions flanking the *Serk3* open reading frames of the potato and tomato homologs and PCR amplified *NbSerk3* homologs from cDNA using Phusion proof reading polymerase (New England Biolabs). Amplicons were cloned into pCRII-BLUNT/Topo (Invitrogen) and sequenced. Sequences were analyzed using Sequencher 4.1 (Gene Codes Corporation). Clones representing both variants (*NbSerk3A*, *NbSerk3B*) were identified based on high overall similarity to SERK3.

### Microbial strains and growth conditions

*A. tumefaciens* GV3101 was used in molecular cloning experiments and was cultured at 28°C in lysogeny broth (LB) media using appropriate antibiotics. DNA was transformed by heat shock into competent *E. coli* TOP10 (Invitrogen) cells or electoporated into electrocompetent *A. tumefaciens*.

The following isolates were used in *Phytophthora* infection assays: *P. infestans* 88069 [39] and a transformant expressing a cytosolic tandem DsRed protein (88069td), *P. infestans* EC1 [40], *P. infestans* T30-4 [41], *P. capsici* LT1534 (http://genome.jgi-psf.org/PhycaF7/PhycaF7.home.html), *P. mirabilis* PIC99114 (collected with other isolates on an excursion published in [42]), *P. palmivora* P16830 from oil palm collected in Occidental, Colombia [43].

### Purification of INF1 protein

INF1 protein was purified as described [44] with modifications. Briefly, *P. infestans* 88069 was grown for 3–4 weeks in plich medium. The culture medium was harvested by filtration and the plich medium was exchanged for 10 mM NaCl with 10 mM Tris HCl (pH 7.4) by dialysis at 4°C. The resulting solution containing INF1 was loaded onto a Fast-Flow Sepharose Q (GE Healthcare) equilibrated column with 10 mM Tris HCl. Subsequently, the column was eluted with a linear gradient of 0–500 mM NaCl in 10 mM Tris HCl (pH 7.4). The presence of INF1 throughout purification was established by silver staining of 15% SDS-PAGE gels. Further analysis of the final pooled fractions by mass spectrometry (data not shown) confirmed the purity of the sample. Concentration was determined by BCA Protein Assay Kit (Thermo Scientific). The protein was further diluted in water before carrying out experiments.

### Infiltration of INF1 protein and cell death scoring

INF1 protein diluted in water was injected into abaxial sides of leaves of 4–5 week old *N. benthamiana* following published protocols [45]. Development of cell death was monitored over time. Cell death was visualised by UV transillumination.

### Measurement of ROS

Generation of reactive oxygen species (ROS) was measured as previously described [46]. Briefly 16 4-mm leaf discs per treatment from 4 week-old wild type *N. benthamiana* leaves were floated 16 h in 200 ul of water in a 96-well plate. Solution was replaced by a luminol/peroxidase mix supplied with either 10 µg/ml INF1 purified protein, 100 ng/ml flg22 peptide or water control. To confirm that the buffer in which INF1 protein was dissolved did not interfere with the ROS measurement, an additional control was included in which flg22 containing solution also had one tenth of such buffer. Luminescence was measured over 380 minutes using an ICCD photon-counting camera (Photek) and analyzed using company software and Microsoft Excel.

### *Phytophthora* spore infections

For *Phytophthora* infection assays, strains were grown on rye sucrose agar as previously described [17] at 18°C in the dark (*P. infestans*, *P. mirabilis*) or on V8 vegetable juice agar plates (*P. capsici*) at 25°C and illumination. *Phytophthora* spores were harvested as described [17], [24]) and diluted to 50.000 spores/ml. Droplets of 10 µl were applied onto abaxial sides of 5 week old detached leaves and incubated for several days on wet paper towels in 100% relative humidity.

### Light Microscopy

Confocal microscopy was used to visualise haustoria of *P. infestans* in cut leaf patches mounted in water, 4 days after transient *A. tumefaciens* mediated expression of plasma membrane localised CFP (pm-CFP) and subsequent infection with *P. infestans* 88069td spore solution. Pictures were acquired on a Leica DM6000B/TCS SP5 microscope (Leica Microsystems CMS GmbH) with Laser settings for CFP (458 nm) and RFP (561 nm).

Mycelial growth of *P. infestans* was visualised using a Leica Stereomicroscope (Leica Microsystems CMS GmbH) mounted with a CCD camera under UV LED illumination and filter settings for DsRed.

### Electron microscopy

Infected leaf samples were cut and immediately placed into fixative (2.5% (v/v) glutaraldehyde in 0.05 M sodium cacodylate, pH 7.3) then placed in a vacuum infiltrator to remove trapped air and to allow the fixative to penetrate all cells. They were then placed in fresh fixative and left overnight at room temperature to fix the tissue. Samples were placed in tissue-handling devices and processed at low temperature by the PLT method, as described [47]. This procedure was followed except for the following modifications; infiltration steps were performed at –20°C with LR White resin plus 0.5% (w/v) benzoin methyl ether, and polymerization was in Beem capsules, with indirect UV irradiation for 24 h at –20°C followed by 16 h at room temperature. The material was sectioned with a glass knife using a Leica UC6 ultramicrotome (Leica). Ultrathin sections of approximately 90 nm were picked up on 200 mesh grids which had been pyroxylin and carbon-coated. Grids were contrast stained with 2% (w/v) uranyl acetate and 2% lead citrate. The grids were viewed in a Tecnai 20 transmission electron microscope (FEI) at 200 kV and images were taken using an AMT XR60B digital camera (Deben).

### *NbSerk3* silencing

We performed gene silencing as described previously [48]. *A. tumefaciens* suspensions expressing the binary TRV RNA1 construct, pTRV1, and the TRV-RNA2 vectors, pTRV::GFP or pTRV::NbSerk3 were mixed in a 2∶1 ratio (RNA1:RNA2) in infiltration buffer medium (10 mM MgCl2, 5 mM 2-N-morpholino ethanesulfonic acid pH 5.3, and 150 µM acetosyringone) to a final OD600 of 0.6. Two week old *N. benthamiana* plants were infiltrated with the Agrobacteria and systemic spread of the virus and system silencing was monitored by morphological changes attributable to *Serk3* silencing and by silencing of two additional plants with TRV::SU that reduced chlorophyll content of silenced leaves.

### RT-PCR

Total RNA of 2 leaf discs per plant was extracted using RNeasy Mini Kit (Qiagen). 3.5 µg RNA were subjected to first strand cDNA synthesis using Revert Aid First Strand cDNA synthesis Kit (Thermo Fisher) and following the manufacturers recommended protocol. Primer combinations NbSerk3-GSPF2/Seb (5′-ATCGCTGGAGGAGTTCCTGCAGG-3′) with NbSerk3-GSPR2/Seb (5′-CAATTGTACCACGTACAGCAGTGGTAAC-3′) and *tubulin* primers were used in a 30-cycle RT-PCR. Amplicon size and intensity was tested on Ethidiumbromid (EtBr) stained agarose gel with UV transillumination.

## Supporting Information

Figure S1 ***NbSerk3*** **variants are silenced by TRV::** ***NbSerk3*** **silencing construct.** RT-PCRs were carried out on *NbSerk3*- or control silenced leaf discs using primers that amplify a region which does not overlap with the silencing target sequence (A). Different amounts of total cDNA were subjected to PCR using control *tubulin* primers or *NbSerk3* specific primers and visualised in an EtBr gel (B).
(TIF)

Figure S2 ***NbSerk3*** **silenced** ***N. benthamiana*** **shows enhanced susceptibility to infection by** ***P. infestans*** **T30-4 and EC1 isolates.** *N. benthamiana* plants were silenced using tobacco rattle virus vectors harbouring green fluorescent protein gene that is not present in *N. benthamiana* (TRV::*GFP*) or a partial *NbSerk3* sequence (TRV::*NbSerk3*). Nineteen days later, leaves were detached and spore droplet inoculated within the dotted lines with *P. infestans* T30-4 (upper row) or EC1 (lower row). Images were taken 6 dpi with UV illumination. Dotted lines represent infected areas.
(TIF)
