# Supplementary figures and images for: The Receptor-Like Kinase SERK3/BAK1 Is Required for Basal Resistance against the Late Blight Pathogen Phytophthora infestans in Nicotiana benthamiana
Source: PLoS One. 2011 Jan 27;6(1):e16608. doi: 10.1371/journal.pone.0016608 (PMC3029390; doi:10.1371/journal.pone.0016608)

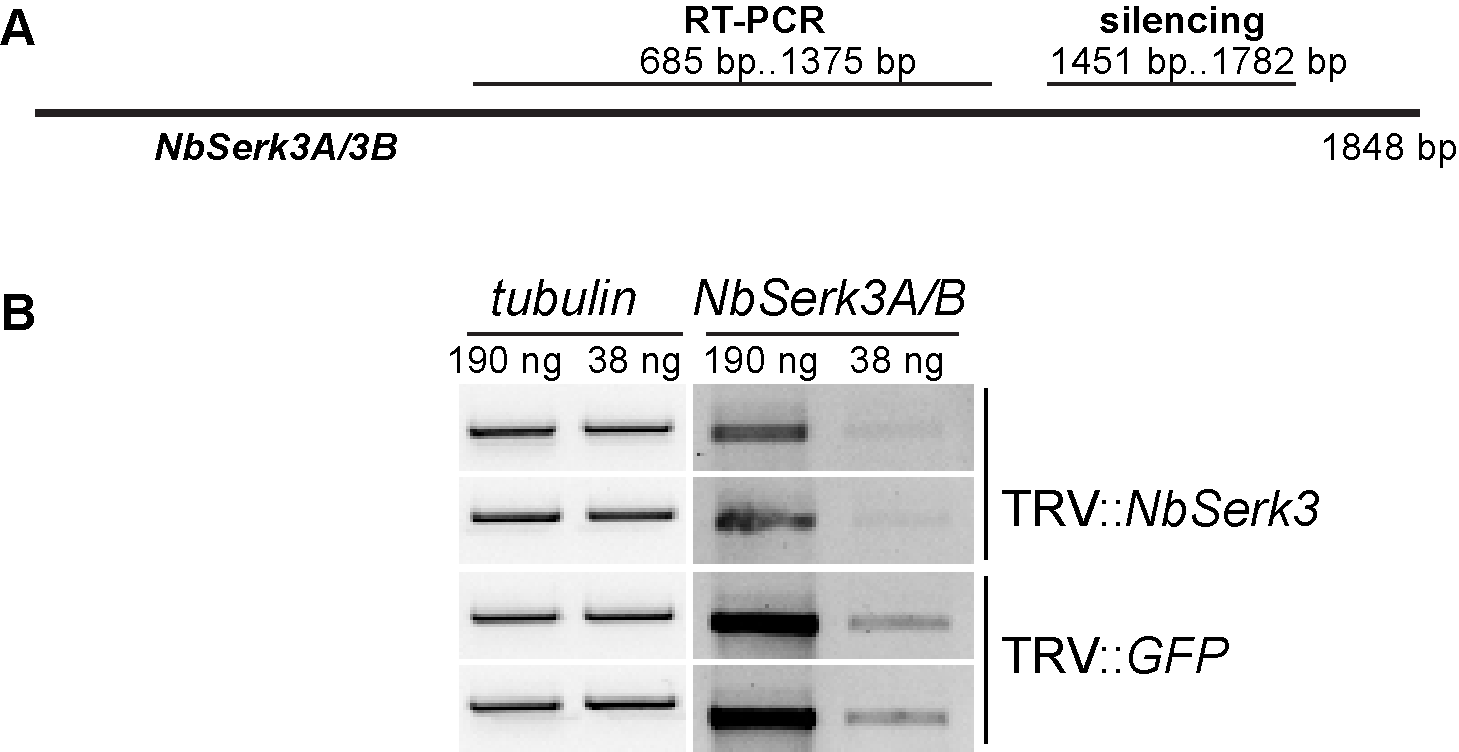

Supplement: Figure S1 — NbSerk3 variants are silenced by TRV:: NbSerk3 silencing construct. RT-PCRs were carried out on NbSerk3- or control silenced leaf discs using primers that amplify a region which does not overlap with the silencing target sequence (A). Different amounts of total cDNA were subjected to PCR using control tubulin primers or NbSerk3 specific primers and visualised in an EtBr gel (B). (TIF) [file pone.0016608.s001.tif]

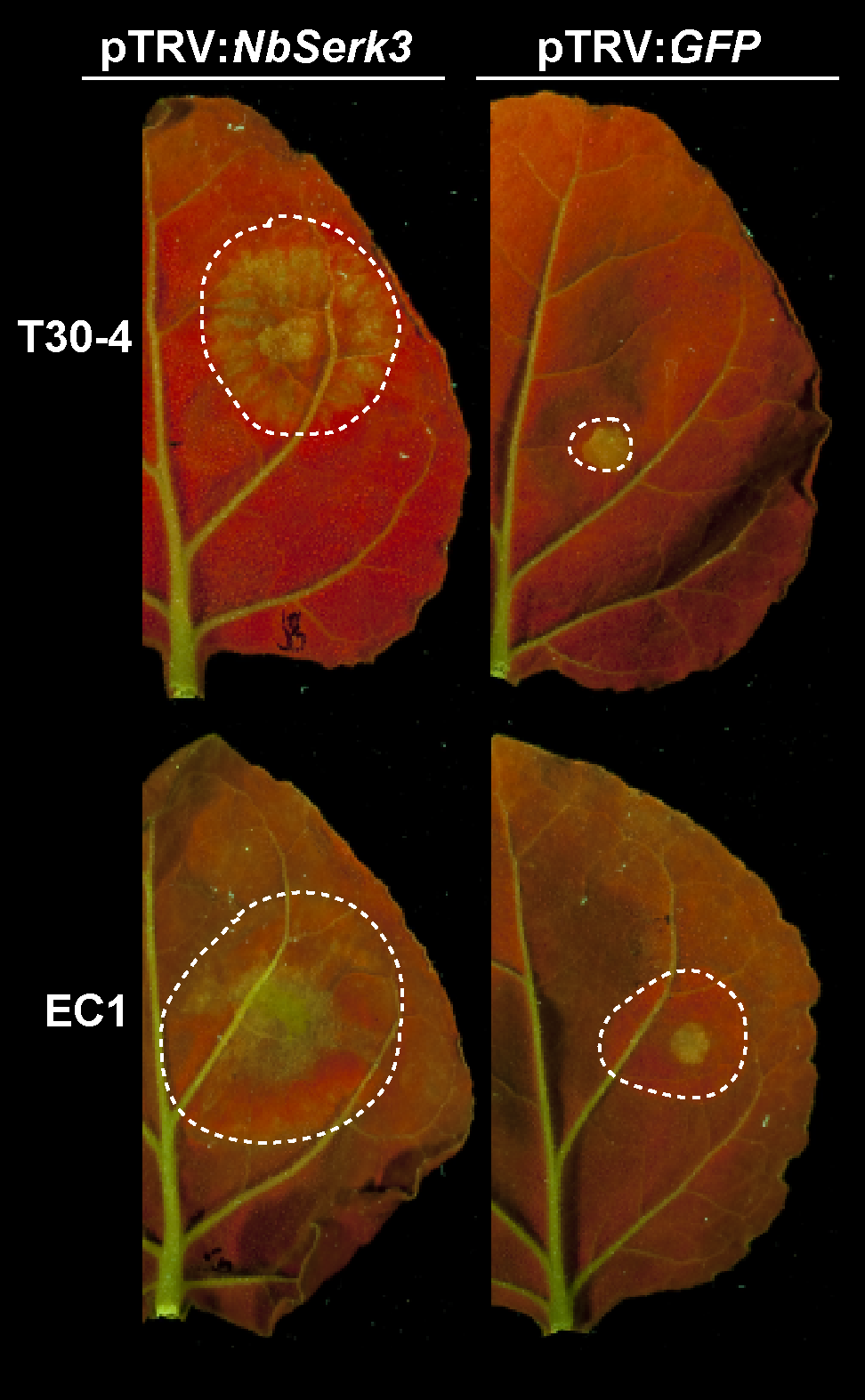

Supplement: Figure S2 — NbSerk3 silenced N. benthamiana shows enhanced susceptibility to infection by P. infestans T30-4 and EC1 isolates. N. benthamiana plants were silenced using tobacco rattle virus vectors harbouring green fluorescent protein gene that is not present in N. benthamiana (TRV::GFP) or a partial NbSerk3 sequence (TRV::NbSerk3). Nineteen days later, leaves were detached and spore droplet inoculated within the dotted lines with P. infestans T30-4 (upper row) or EC1 (lower row). Images were taken 6 dpi with UV illumination. Dotted lines represent infected areas. (TIF) [file pone.0016608.s002.tif]
